# From evolving artificial gene regulatory networks to evolving spiking neural networks for pattern recognition

**DOI:** 10.1186/1471-2202-14-S1-P423

**Published:** 2013-07-08

**Authors:** Ahmed Abdelmotaleb, Maria Schilstra, Neil Davey, Volker Steuber, Borys Wróbel

**Affiliations:** 1 Biocomputation Research Group, University of Hertfordshire, Hatfield, Herts AL10 9AB, UK; 2 Evolutionary Systems Laboratory, Adam Mickiewicz University, Poznan, 61-712, Poland; 3 Systems Modeling Laboratory, IOPAN, Sopot, 81-712, Poland

## 

The GReaNs platform (which stands for Gene Regulatory evolutionary artificial Networks) is an artificial life software platform that has been previously shown to allow the evolution of networks for signal processing and animat control [1]. The structure of the resulting network in this model is encoded in a linear genome, without imposing any restrictions on the size of the genome or the size of the network. We extended this model to encode spiking neural networks [4]. In previous work using GReaNs [1], each node in the artificial network has been considered to be an analog of a biological transcriptional unit. However, the nodes can also be seen as artificial neurons. Our extension has introduced to GReaNs two models of spiking neurons (LIF: leaky integrate and fire neurons with a fixed threshold [2] and AdEx: adaptive-exponential integrate and fire [3]). Here we use GReaNs for the evolution of spiking networks for pattern recognition. The genetic algorithm used in GReaNS is based on evolving the number of the neurons and the weights of the connections, but the synaptic delays are constant. We evolved a network that generates spikes at a maximum firing rate when the network is presented with one spike from five input neurons in a specific order, and that stays silent when the order is reversed. We also used three inputs, each with one spike, requiring the network to fire at a maximum rate for a specific temporal order of spikes and to remain silent for all the other permutations. In further work, we plan to investigate the generalization abilities of the evolved networks and the robustness of their behaviour to noise. GReaNs can export a PyNN script, which allows the simulation of an evolved network on different backends, including neuromorphic real-time hardware for real time simulation.
